# Still Birth classification: Application of Relevant Condition at Death (ReCoDe) classification system in a tertiary care hospital of Pakistan

**DOI:** 10.12669/pjms.38.1.4470

**Published:** 2022

**Authors:** Urooj Kashif, Shelina Bhamani, Asma Patel, Zaheena Shamsul Islam

**Affiliations:** 1 Dr. Urooj Kashif, Senior Instructor and Urogynecologist, Department of Gynecology/Obstetrics, Aga Khan University Hospital, Karachi, Pakistan. Department of Gynecology/Obstetrics, Aga Khan University Hospital, Karachi, Pakistan; 2 Dr. Shelina Bhamani Assistant Professor Clinical Research, Department of Gynecology/Obstetrics, Aga Khan University Hospital, Karachi, Pakistan; 3 Dr. Asma Patel, Senior Resident, Department of Gynecology/Obstetrics, Aga Khan University Hospital, Karachi, Pakistan; 4 Dr. Zaheena Shamsul Islam Assistant Professor, Department of Gynecology/Obstetrics, Aga Khan University Hospital, Karachi, Pakistan

**Keywords:** Stillbirth, ReCoDe, intrauterine demise, developing world

## Abstract

**Objectives:**

To determine the cause of stillbirth after application of relevant condition at death (ReCoDe) classification system.

**Methods:**

This was a retrospective cross sectional study of 207 women diagnosed with stillbirth after 24 completed weeks of pregnancy at the Aga Khan University Hospital (AKUH), Karachi between 1st January 2015 and 31st December 2019. The primary objective was to find the cause of stillbirth according to the new classification of relevant condition at death (ReCoDe).

**Results:**

There were a total of 32413 live births and 207 stillbirths during the study period thus stillbirth rate of 6 per 1000 live births. In this study, 80% of women were in the age group of 20-35 years, 16% had advanced maternal age while 3.8% of women accounted for less than 20 years. Among the maternal factors; 54.5% cases were booked and the remaining were were un-booked cases. Pre-eclampsia was the most common associated maternal condition (14.9%).

Fetal cause accounted for 34.7% of stillbirths and the fetal growth restriction (FGR) was the most common; 23.6%. After application of ReCoDe classification, in 81% of stillbirth cases associated condition were found and only 18.8% of cases were categorized unexplained.

**Conclusion:**

Application of ReCoDe classification is easy to understand and applicable, especially in low resource settings with associated causes identified in vast majority of cases.

## INTRODUCTION

Stillbirth is defined as the intrauterine demise (IUD) of a fetus after the age of viability. Though the World Health Organization (WHO) recommendations for reporting stillbirth is fetal birthweight of > 1000 grams, or gestational age of 28 weeks’ gestation or more, or when the body length of fetus is 35 cm or more,^1^,^2^ there is wide variation around the globe to define the age of viability of a fetus, as the survival is dependent on access to and utilization of antenatal care, presence of skilled birth attendants and increased attention to known maternal risks for stillbirth. Hence, in the United States the age of viability is 20 weeks or more and in Pakistan it is 24 weeks or later.^3^

Stillbirth is one of the most common adverse pregnancy outcome and largest contributor to perinatal mortality. About 2.6 million stillbirth were reported in 2015,^1^ with 98% occurring in the low and middle income countries.^4^ Pakistan has one of the highest stillbirth rate in the world. A recent study from a tertiary care hospital in Karachi revealed a stillbirth rate of 18 per 1000 births which is relatively lower than published data from Pakistan.^5^

There are many causes of still birth and different classification systems have been proposed in an attempt to understand the underlying factors and events leading to stillbirth but about two thirds of stillbirths are being classified as unexplained by the current classification systems.^6^

Classification of stillbirths by Relevant Condition at Death (ReCoDe) is one of the only classification systems specifically developed to identify causes of fetal death-”What went wrong, not necessarily why”.^7^,^8^ It is derived from a population-based cohort study in England, and using this system, nearly 85% of stillbirths can be classified.^9^ It is structured, in a hierarchial system, first addressing conditions affecting the fetus and then moving in simple anatomic groups, subdivided into pathophysiologic conditions where the first on list is the primary condition applicable to a case.^8^

This classification relies more on clinical information rather than histopathologic data, it is more relevant in the developing world where lack of expertise and unwillingness of parents for autopsy and/or placental biopsy is not a routine practice. In Aga Khan University Hospital (AKUH), all stillbirths are registered in the departmental data. However, no classification system of stillbirths is used and hence comparison with national and international data is not possible. Therefore, we performed this study with the aim to determine the cause of stillbirth rate by applying the ReCoDe classification system of stillbirth to our population. This would help the physician in counseling their patients about causes of stillbirths in our setup, and the use of preventive and screening strategies to reduce the risk of stillbirth.

## METHODS

We conducted a cross sectional study of 207 women at the Aga Khan University Hospital (AKUH), Karachi between 1st January 2015 and 31st December 2019. We included all pregnancies complicated by stillbirth after 24 weeks of gestation. All women who delivered outside AKUH, or where the delivery or stillbirth information were missing were exclude.

Variables like demographic data, maternal characteristics including maternal age, gestational age at diagnosis, fetal weight at delivery, and booking status and maternal information regarding pre-eclampsia, diabetes, thyroid dysfunction was collected on a structured Performa from medical record files. Similarly findings of the ultrasound reports and other investigations, the information of stillborn baby, placenta and umbilical cord examination after delivery were recorded.

Data was entered and analyzed in SPSS package version 21 (IBM Corp.; Armonk, NY, USA). Frequency and percentages were calculated for these categorical variables. The study was conducted after approval from hospital ethical review committee (ERC- 2019-1962-5417).

## RESULTS

There were total 207 cases of stillbirth during the study period fulfilling the inclusion criteria. Total live births during this duration were 32413 making six stillbirths per 1000 live births.

Basic demographics are shown in Table-I. The age distribution of women with in utero fetal demise showed that majority (80%) of women were in the age group of 20-35 years. Advanced maternal age accounted for 16% of cases while 3.8% of women were less than 20 years. 54.5% cases were booked hospital cases while almost 45.4% were un-booked cases which points towards a high referral to the tertiary care setup.

**Table I:** Demographics.

Variable	n=207 n (%)
***Age (years)***	
<20	8 (3.9)
20-35	166 (80.2)
>35	33 (15.9)
***Gestational age (weeks)***	
<28	55 (26.6)
28-31.6	51 (24.6)
32-35.6	52 (25.1)
36-39.6	41 (19.8)
>40	8 (3.9)
***Booking status***	
Booked	113 (54.6)
Un booked	94 (45.4)
***Fetal weight (grams)***	
<500	18 (8.7)
500-999	66 (31.6)
1000-1499	24 (11.6)
1500-1999	29 (14.0)
2000-2499	28 (13.5)
>2500	39 (18.8)

Data are presented as numbers and percentages.

The application of ReCode classification system on stillbirths is shown in Table-II. 34.7% of stillbirths were attributed to the fetal causes with the largest group comprised of fetal growth restriction (23.6%). Maternal causes with 30.4% of stillbirths, among which the most common associated condition was pre-eclampsia. The group where no other cause could be found was categorized as unclassified and comprised of 18.8% of cases.

**Table II:** Classification of stillbirths by ReCoDe.

Conditions	n=207 (%)
Fetal causes	72 (34.7%)
Congenital abnormalities	13 (6.2)
Hydrops fetalis	4 (1.9)
Fetal growth restriction	49 (23.6)
Infection	1 (0.48)
Selective intrauterine growth restriction in twin pregnancy	5 (2.4)
Co-twin intrauterine death	5 (2.4)
Rh immunization	2 (0.9)
Umbilical cord causes	1 (0.48%)
Cord entanglement	1(0.48)
Placental causes	6 ( 2.8)
Placental abruption	6(2.8)
Amniotic fluid causes	9(4.3%)
Chorioamnionitis	2 (0.9)
Polyhydromnios	1(0.48)
PPROM	6 (2.8)
Uterine causes	6(2.8%)
Uterine rupture	6 (2.8)
Maternal causes	63(30.4%)
Diabetes mellitus	12 (5.7)
Pre-eclampsia/eclampsia	31 (14.9)
APLA/thrombophilia	6 (2.8)
Cardiac disease	1 (0.48)
Maternal sepsis/infection	6 (2.8)
Hypothyroidism	2 (0.9)
HELLP	3 (1.4)
Peri-partum cardiomyopathy	1 (0.48)
Status epilepticus	1 (0.48)
Intra partum causes	2(0.9)
Asphyxia	2 (0.9)
Trauma related causes	2(0.9%)
External trauma	2 (0.9)
Unclassified	39 (18.8%)

*Abbreviations;* APLA (Antiphospholipid antibodies syndrome) HELLP (hemolysis, elevated liver enzymes, low platelets) PPROM (preterm pre-labour rupture of membranes).

Data are presented as numbers and percentages.

## DISCUSSION

Individual research studies list various reasons of stillbirth in urban populations of Pakistan. The causes range from antepartum maternal disorders and intrapartum asphyxia to unexplained antepartum and intrapartum causes in national surveys conducted in Pakistan between 2006 and 2013. Though a standard stillbirth classification model is not in effect. In our study the use of a classification system allowed us to identify the associated conditions at the time of stillbirth, which may be contributory to the occurrence of fetal demise.

Being a tertiary care hospital, 46% of our cases were un-booked and referred. This is due to the fact that a vast majority of women don’t have access to the antenatal care. According to a recent study only 57% of pregnant Pakistani women had attended recommended four or more antenatal visits.^10^ Seeking antenatal health care was even less in the first trimester of pregnancy, a visit most aimed at identification of high risk factor and thus provision of quality care according to the woman’s need. Failure to access healthcare in early pregnancy and identification of risk factors in these vulnerable women seem to be the major reason of high stillbirth rate in Pakistan. In another study conducted by Aleha Aziz et al. highlighted the insufficient human and material resources available to Pakistani women when compared to other low resource countries.^11^

We also observed our data for the trends in stillbirth in relation to gestational and found uniformly high rate across all gestational ages. The data across the globe is however, not uniform. World Health Organization statistic show the number of stillbirths has declined by 19.4% between 2000 and 2015 around the globe, yet three fourth of the stillbirth occur in the Southeast Asia and sub-Saharan Africa, secondary to absence of skilled health professional birth attendants.^12^ This resultant half of stillbirth occur intrapartum. In regions with high stillbirth rate, it mirrors the inability of women to access health care, and thus lack of identification, investigation and intervention result in loss of preventable stillbirth.^13^

Our study showed that after application of classification of stillbirth by relevant condition at death (ReCoDe) identified causes in 81% cases while only 19% remained unidentified. Unexplained stillbirth is where no identifiable fetal, placental, maternal, or obstetric etiology could be found, when sufficient information is not available or when the current diagnostic ability cannot establish the cause of stillbirth.^14^ Although different classification systems are currently in use to help in identification the cause of stillbirth, Gardosi et al. applied and compared different classification systems to a large cohort and found the ReCoDe system to be a better performing system, as it resulted in the assignment of a relevant condition in 85% cases.^8^ Similarly, in another Indian study, application of recode resulted in 87.5% cases to be classified according to the associated condition and only 13.5% were left unexplained.^15^ Our results are consistent with the results of these researchers, suggesting use at a wider level as more heath resources can be allocated to focus on areas of concern.

The most common condition found associated at the time of stillbirth was fetal growth restriction (FGR) present in 23.6% of cases. FGR is an established risk factor for stillbirth with highest risk with severe growth restriction and lower percentiles. In a study by Ruth C Fretts, it was found that the odd ratio for stillbirth was 11.8 for a small for gestational age fetus in comparison to an appropriate for gestational age fetus.^16^ Therefore, timely detection of FGR will provide an opportunity for interventions to reduce the morbidity and mortality associated. Gardosi in his work has shown that antenatal identification of FGR can reduce the rate of stillbirth.^17^

The risk of fetal growth restriction is even higher in twin pregnancies. This is also because in the presence of selective fetal growth restriction, the parents may choose to advance the pregnancy even with imminent fetal death in one of the fetuses as the best interest of fetuses may be at odds with each other.^18^ In our study, 2.4% of the stillbirths in twins were due to selective fetal growth restriction. This proves FGR to be one of the major cause of stillbirth.

The current study shows that 18% of pregnancies with FGR also had co-existent preeclampsia. This is due to the fact that both conditions share a common underlying pathophysiology.^19^ Preeclampsia alone was found in 14% of stillbirth cases. This is in agreement with QE Harmon’s findings, where they noted that risk of fetal death begins with apparent preeclampsia.^20^

The underlying factors of stillbirths are multiple and diverse, including avoidable and unavoidable causes. These are related to individual behaviours, knowledge, attitudes and practices, societal and cultural values, healthcare providers’ knowledge, attitudes and practices and quality of healthcare. In our data, the majority were attributable to cause where preventive strategies can greatly affect outcomes, with significant reduction in the stillbirth rate.

A local study at a tertiary university hospital in Pakistan showed that majority of stillbirths were due to risk factors which could be identified in the antenatal period. According to this study obstructed labour, hypertensive disorders, abruptio placentae, placenta previa and preterm labour were significantly associated with stillbirth.^21^

In Pakistan, stillbirth remains poorly reported. In addition, lack of use of any classification system result in inability to identify the cause or contributory factor leading to stillbirth. Application of ReCoDe classification is easy to understand and applicable especially in low resource settings with associated causes identified in vast majority of cases. This is also helpful in counseling the parents who are seeking explanations and are trying to come to terms with the loss; the clinicians who are seeking to advise the mother on the implications and plans for future pregnancies; the health care institutions which need to review the service they are providing; and the planners and commissioners who are seeking to improve the service.

### Strength and limitations of the study:

The strength of our study is long time duration spanning over six years. The limitation is its retrospective nature, small number of cases and single hospital based.

## CONCLUSIONS

ReCoDe is an ideal stillbirth classification system for low resource setting to develop focused preventive and screening strategies for the reduction of still birth rate.

### Author’s Contribution:

**ZSI:** Conceived the idea, manuscript preparation and drafting. She is also responsible for integrity and accuracy of the work.

**UK:** Data collection and statistical analysis, Manuscript preparation and drafting review.

**SB:** Manuscript preparation, study design, methodology.

**AP:** Data collection.
